# A comparison of the clinical efficacy of total hip arthroplasty via direct anterior approach and posterior approach: A meta-analysis

**DOI:** 10.1097/MD.0000000000039237

**Published:** 2024-08-09

**Authors:** Hao Wang, Jin-Feng Liu, Fengjing Wang, Tiange Yuan, Hengduo Jiang, Zhuoqi Wei, Yang Zhang, Jiahao Meng

**Affiliations:** a Xiangya School of Medicine, Central South University, Changsha, China; b Department of Oncology, Rizhao Hospital of Traditional Chinese Medicine, Rizhao, China; c Weifang Medical University, Weifang, China; d Xinjiang Medical University, Urumqi, China; e Department of Orthopaedics, Xiangya Hospital, Central South University, Changsha, China.

**Keywords:** direct anterior approach, meta-analysis, posterior approach, total hip arthroplasty

## Abstract

**Background:**

The approach of total hip arthroplasty (THA) has long been controversial, and many studies have compared different approaches. However, there is still a lack of consistent conclusions and comprehensive, systematic comparisons and evaluations.

**Methods:**

This study retrieved 7 databases: PubMed, Web of Science, Embase, Cochrane Library, Chinese Biomedical Literature Database, China National Knowledge Infrastructure, and Wanfang Database. The search time ranged from the establishment of each database to November 1, 2023. Data analysis was performed using Review Manager 5.4, and outcome was presented as the weighed mean difference for continuous data and risk/odds ratio for dichotomous data. We used the Mantel–Haneszel method and random effects model to obtain the overall effects of the differences in the impact of 2 surgical methods on clinical outcomes in all included studies.

**Results:**

A total of 33 articles were included in this study, including 14478 participants, 4911 participants in DAA group and 9567 participants in PA group. The visual analogue scale scores of the DAA group at 1 day and 2 days after THA were significantly lower than those of the PA group (mean difference [MD] = −0.56, 95% confidence interval [CI]: −0.83 to −0.30, *P* < .01) at 1 day and (MD = −0.67, 95% CI: −1.16 to −0.17, *P* = .01) at 2 days. The risk of intraoperative fracture (odds ratio = 2.18, 95% CI: 1.11–4.29, *P* = .05) and lateral femoral nerve injury (risk ratio = 7.84, 95% CI: 1.69–36.42, *P* < .01) in the DAA group was significantly higher than that of the PA group. The number of prostheses in the Lewinnek safe zone of the DAA group was significantly higher than that of the PA group (risk ratio = 1.13, 95% CI: 1.00–1.27, *P* = .05). The results showed no significant difference between the DAA group and the PA group in the time to stop using walking aids, dislocation rate, groin pain, incision complications, heterotopic ossification, intraoperative blood loss, and acetabular anterior (*P* > .05).

**Conclusion:**

Compared with the PA group, patients in the DAA group showed more ideal anatomical and imaging results, shorter hospital stay, and showing advantages in postoperative pain, but with a higher incidence of intraoperative complications.

## 1. Introduction

Total hip arthroplasty (THA) is the replacement of damaged femoral head and acetabulum with artificial hip joints to restore hip joint function and weight-bearing. It was previously believed that severe hip osteoarthritis, hip dysplasia, severe or elderly femoral neck fractures, traumatic arthritis and bone tumors were indications for this surgery. With the continuous development of medical technology, the age range and indications for this surgery have gradually expanded, with over 500,000 procedures performed annually worldwide.^[1]^ THA not only relieves hip joint pain and improves its therapeutic purpose but also maintains joint stability, equalizes limb length, and improves quality of life and joint mobility.^[2]^ Common surgical approaches for THA include the direct anterior approach (DAA), posterior approach (PA), lateral approach, and anterolateral approach.^[3]^

PA is one of the most commonly used THA surgical approaches globally.^[4]^ However, an increasing number of clinical studies have shown that PA exposes limited postoperative pain relief, has higher postoperative complications, inflicts significant damage to surrounding tissues, and exhibits slow long-term recovery rates.^[5,6]^ In comparison to PA, DAA, a modification of the Heuter approach,^[7]^ enters the hip joint through the muscle gap between the tensor fasciae latae, sartorius, and rectus femoris, thereby protecting the soft tissues around the hip and aiding in maintaining joint stability.^[8]^ DAA also offers advantages such as less bleeding and faster postoperative recovery.^[9]^ Nevertheless, some scholars have pointed out that DAA surgical techniques are not yet mature and do not exhibit significant advantages in postoperative functional recovery and acetabular prosthesis positioning.^[10,11]^ Moreover, complications such as injury to the superior gluteal nerve are more likely to occur.^[12]^ Scholars have conducted several meta-analyses on DAA and PA in the past,^[6,13,14]^ but the results are still controversial and cannot draw definitive conclusions. We also found that previous related studies have limited indicators for measuring clinical outcomes. Therefore, we hope to combine recent clinical studies to comprehensively compare these 2 mainstream surgical methods from as many perspectives as possible, providing high-level evidence-based medicine evidence and reference for surgical selection in different clinical contexts.

## 2. Materials and methods

### 2.1. Literature search

The literature search for this study was independently conducted by 2 researchers using the same search strategy, and the results were integrated by a third researcher. Six databases were searched: PubMed, Web of Science, Embase, Cochrane Library, China National Knowledge Infrastructure, and Chinese Biomedical Literature Database. The search was conducted from the inception of each database to November 1, 2023, with no language restrictions. The English search utilized MeSH terms, including: DAA, PA, and THA.

### 2.2. Inclusion criteria

Study participants: patients undergoing primary THA; Intervention: studies reporting outcomes of patients undergoing THA with DAA or PA; Study design: randomized controlled trials (RCTs), retrospective cohort studies, and prospective cohort studies; Outcomes: articles reporting postoperative functional assessment indicators, intraoperative and postoperative complications, and indicators of patient postoperative recovery.

### 2.3. Exclusion criteria

Interventions other than DAA or PA; studies solely reporting either DAA or PA intervention without comparison between the two; sample size of either DAA or PA group less than 20; and duplicate publications, case reports, reviews, autopsy studies, or animal studies.

### 2.4. Data extraction

Two researchers independently conducted data extraction using a standardized Excel spreadsheet, followed by cross-verification of the extracted results. In case of discrepancies, a third researcher reviewed the original text for reassessment. Variables extracted in this study included: basic study characteristics: title, first author, publication year, study type; indicators of postoperative recovery: postoperative visual analogue scale (VAS) score, time to discontinue walking aid postoperatively; indicators of intraoperative and postoperative complications: intraoperative fractures, postoperative dislocations, lateral femoral cutaneous nerve injury, groin pain, incision complications, heterotopic ossification, operative time, length of hospital stay, intraoperative blood loss; and radiographic indicators: acetabular anteversion angle, acetabular abduction angle.

### 2.5. Quality assessment of included studies

Two researchers independently conducted quality assessment of included studies, followed by cross-verification of the assessment results. Any discrepancies were reviewed by a third researcher. Quality assessment of RCTs was performed using the Jadad scoring system, which evaluates randomization, blinding, and follow-up, with a total score of 5. Scores of 0 to 2 indicate low-quality studies, while scores of 3 to 5 indicate high-quality studies. The Newcastle Ottawa Quality Assessment Scale was used to assess the quality of retrospective and prospective cohort studies, which includes 3 aspects: selection of cohorts, comparability, and ascertainment of outcomes, with a total of 9 stars. More stars indicate higher quality: 0–3 stars: low quality, 4–6 stars: moderate quality, and 7–9 stars: high quality.

### 2.6. Statistical analysis

For dichotomous outcomes, the Mantel–Haenszel method was used to calculate the risk ratio (RR) as the effect measure for meta-analysis. For continuous outcomes, the weighted mean difference (MD) was used as the effect measure for meta-analysis. The *I*^2^ statistic was used to assess statistical heterogeneity among included studies. If significant statistical heterogeneity existed among the included studies (*I*^2^ > 50%), a random-effects model was used to pool effect measures; if there was no significant statistical heterogeneity among the included studies (*I*^2^ < 50%), a fixed-effects model was used to pool effect measures. The Clopper–Pearson exact method was used to calculate the 95% confidence interval (CI) for effect measures. Forest plots were used to display the results of the meta-analysis. Statistical analysis was performed using Review Manager 5.4. A two-tailed test was used, and a *P*-value less than .05 was considered statistically significant. We used *Q*-tests and *I*^2^ statistics to test for heterogeneity in each study. And DerSimonian-Laird estimator was used to calculate the heterogeneity variance (Tau^2^).

## 3. Results

### 3.1. Literature search results

**Figure 1.**
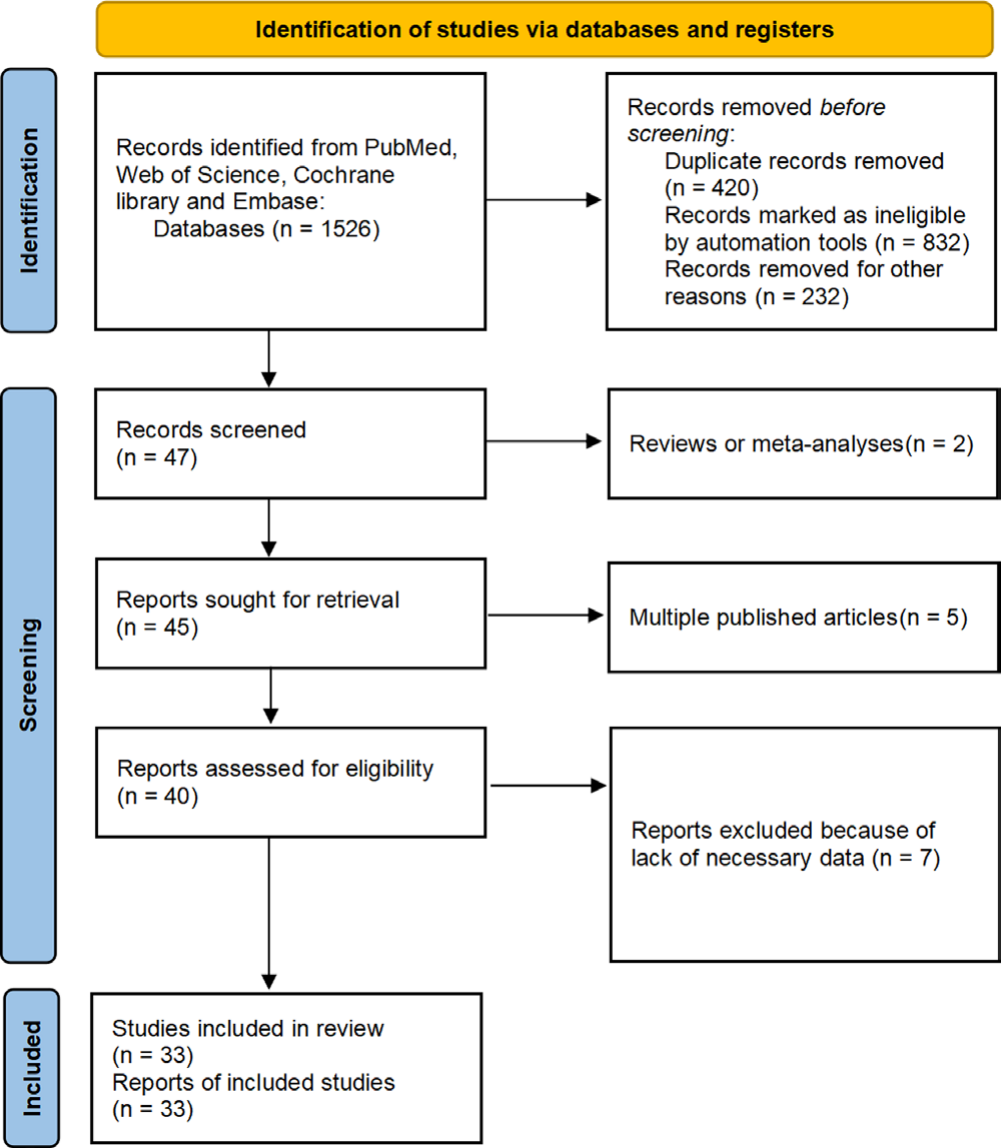
Flow diagram-Literature inclusion and exclusion process. A total of 1526 original literature were retrieved in this study, and 1484 studies that did not meet the inclusion and exclusion criteria were excluded by reading the title and abstract. A preliminary screening of 47 literature was conducted. After reading the entire text, 7 articles lacking the necessary research data were further excluded, 5 were published repeatedly, and 2 were review articles. A total of 33 articles were ultimately included.

A total of 1526 original articles were identified in the initial literature search. After screening titles and abstracts, 1484 studies were excluded for not meeting the inclusion and exclusion criteria of this study. 33 articles were finally included in the analysis. The flowchart depicting the literature inclusion and exclusion process is presented in Figure 1. The basic characteristics of the included studies are summarized in Table 1.

**Table 1:** Characteristics of included studies.

Author	Type	Sample size	Gender (male/female)		Age (*x̄±*SD)		BMI (*x̄±*SD)		Follow-up time	Assessment
		DAA/PA	DAA	PA	DAA	PA	DAA	PA		
Bergin 2011^[15]^	Prospective cohort	29/28	10/19	14/14	68.8 ± 9.1	65.1 ± 11.3	26.3 ± 5	27.8 ± 5	2 d	8^†^
Spaans 2012^[16]^	Retrospective study	46/46	24/22	14/32	69	68	25	29	1 yr	8^†^
Barrett 2013^[13]^	RCT	43/44	29/14	19/25	61.4 ± 9.2	63.2 ± 7.7	30.7 ± 5.4	29.1 ± 5	1 yr	3^*^
Martin 2013^[17]^	Prospective cohort	41/47	14/27	21/26	63	57	28.5	34.1	6 mo	7^†^
Nam 2013^[18]^	Retrospective study	110/110	29/71	45/65	66.7 ± 11.9	66.8 ± 11.1	28.3 ± 5.8	27.4 ± 5.9	6 wk	7^†^
Schweppe 2013^[19]^	Retrospective study	100/100	46/54	53/47	61 ± 1.1	62 ± 1.3	29.1 ± 0.8	31.3 ± 0.7	Unknown	7^†^
Rathod 2014^[20]^	Retrospective study	286/293	130/156	126/167	61.8 ± 12	60 ± 11	26.4 ± 5	25.9 ± 4	30 mo	8^†^
Rodriguez 2014^[21]^	Prospective cohort	60/60	28/32	26/34	60 ± 10	59 ± 6	27 ± 4	28 ± 4	1 yr	9^†^
Taunton 2014^[14]^	RCT	27/27	12/15	13/14	62.05	66.4	27.7	29.2	1 yr	4^*^
Zawadsky 2014^[22]^	Retrospective study	50/50	22/28	14/36	60.8 ± 11.8	56 ± 11.4	28.6 ± 6.2	27.9 ± 6.2	6 wk	8^†^
Christensen 2015^[6]^	RCT	28/23	13/15	11/12	64.3 ± 9.1	65.2 ± 9.1	31.5 ± 5.1	30.4 ± 3.6	6 wk	4^*^
Hamilton 2015^[23]^	Retrospective study	100/100	61/39	64/36	62.5	61.1	28.8	29	2 yr	8^†^
Poehling-Monaghan 2015^[24]^	Retrospective study	126/96	59/67	52/44	64.8 ± 12.4	63.9 ± 12.5	30 ± 5.5	30.5 ± 6	2 mo	8^†^
Balasubramaniam 2016^[25]^	Retrospective study	50/42	30/30	14/28	62.5	57	31.3	29.9	Unknown	9^†^
Malek 2016^[26]^	Retrospective study	265/183	117/148	86/97	70.8	70	28.5	29	18 mo	7^†^
Maratt 2016^[27]^	Retrospective study	2147/2147	978/1169	1030/1117	64.36 ± 10.93	64.84 ± 12.08	28.97 ± 5.51	29.3 ± 5.01	3 mo	8^†^
Tripuraneni 2016^[28]^	Retrospective study	66/66	26/40	26/40	60.2	60.2	27.6	27.8	2 yr	8^†^
Luo 2016^[29]^	RCT	52/52	17/35	22/30	61.5 ± 7.2	63.7 ± 6.8	22.7 ± 4.4	24.1 ± 3.7	14 mo	3^*^
Shi 2016^[30]^	Retrospective study	85/86	47/38	49/37	62.3 ± 19.8	61.9 ± 21.8	24.3 ± 8.5	24.9 ± 9.1	1 yr	8^†^
Cheng 2017^[31]^	RCT	35/38	15/20	18/20	59	62.5	27.7	28.3	3 mo	4^*^
Poehling-Monaghan 2017^[32]^	Prospective cohort	50/50	26/24	22/28	63 ± 9.3	63 ± 11.3	31 ± 5.11	30 ± 8.09	2 mo	8^†^
Rykov 2017^[33]^	RCT	23/23	8/15	11/12	62.8 ± 6.1	60.2 ± 8.1	29 ± 5.6	29.3 ± 4.8	6 mo	4^*^
Zhao 2017^[34]^	RCT	60/60	24/36	26/34	64.88 ± 12.13	62.18 ± 14.72	24.35 ± 3.1	25.58 ± 2.83	6 mo	4^*^
Ponzio 2018^[35]^	Retrospective study	289/4249	122/167	1900/2349	65.1 ± 9.8	64.7 ± 11.2	28.4 ± 5.5	28.1 ± 5.7	4 yr	8^†^
Zhang 2018^[36]^	Retrospective study	35/48	22/13	31/17	49.6	51.2	25.46 ± 3.82	26.73 ± 4.32	44 mo	7^†^
Hart 2019^[37]^	Prospective cohort	293/1109	124/169	522/587	64.3 ± 10.9	62.4 ± 14	29.9 ± 5.3	30.7 ± 6.8	1 mo	8^†^
Nelms 2020^[38]^	Retrospective study	34/28	15/20	21/13	61.4 ± 8.1	61.2 ± 11	26.8 ± 4.9	30.1 ± 4.9	4 mo	9^†^
Ma 2020^[39]^	Retrospective study	150/150	78/72	71/79	57.95 ± 3.72	58.24 ± 4.68	24.76 ± 1	24.53 ± 1.06	3 mo	7^†^
Yang 2022^[40]^	Retrospective study	31/31	12/19	10/21	60.31 ± 6.57	60.85 ± 6.78	26.54 ± 4.52	26.98 ± 5.51	6 mo	8^†^
Young-Yool Chung 2022^[41]^	Retrospective study	36/31	3/33	5/36	78.19 ± 9.44	76.45 ± 6.79	22.60 ± 4.26	22.18 ± 5.81	Unknown	8^†^
Zhao Wang 2022^[42]^	Retrospective study	47/33	25/22	19/14	65.2 ± 4.4	64.7 ± 5.2	23.5 ± 1.1	23.2 ± 1.4	6 mo	8^†^
Xin Jin 2023^[43]^	RCT	53/53	28/25	22/31	51.4 ± 13.6	52.3 ± 12.6	21.8 ± 2.2	21.9 ± 2.8	2 yr	4^*^

* Jadad score.

† NOQAS score.

The quality assessment results of the included literature in this study are as follows: Among the 33 articles included, 8 were RCTs,^[16,18,19,29,31,33,34]^ and 25 were non-RCT studies.^[15,17,20–28,30,32,35–42]^ A total of 14,478 patients were included, with 4911 in the DAA group and 9567 in the PA group.

### 3.2. Results of literature quality assessment in section

Among the 8 RCT studies, 2 had Jadad scores of 3, while the remaining RCT studies had scores of 4. Among the 21 non-RCT studies included in this study, the lowest Newcastle Ottawa Quality Assessment Scale score was 7, with 6 non-RCT studies rated as 7, 16 as 8, and 3 as 9. This indicates that the quality of the literature included in this study is relatively high. Specific scoring details are presented in Table 1.

### 3.3. Systematic review and meta-analysis results

#### 3.3.1. Postoperative functional indicators

**Figure 2.**
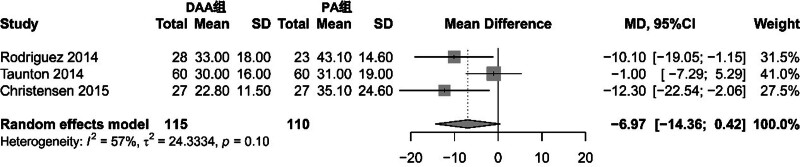
Forest plot comparing the time to stop using assistive devices after surgery between the DAA group and the PA group. There are 3 articles reporting the time for patients to stop using walking aids after surgery. The results showed that the PA group had a longer time, but the inter group difference was not significant (MD = −6.97, 95% CI: −14.36 to 0.42, *P* = .06). CI = confidence interval, DAA = direct anterior approach, MD = mean difference, PA = posterior approach.

Thirteen articles reported results on postoperative functional recovery.^[15–17,21,25,27,29–31,33,37,40]^ Among them, 3 articles reported the time patients stopped using walking aids postoperatively.^[31,33,40]^ The meta-analysis results showed that the PA group had a longer time, but the difference between groups was not significant (MD = −6.97, 95% CI: −14.36 to 0.42, *P* = .06). See Figure 2 for specifics. Other postoperative functional recovery indicators were not suitable for meta-analysis due to differences in follow-up time and assessment indicators; therefore, this study conducted descriptive analyses on these indicators.

Cheng et al found no difference in Oxford Hip Score and The Western Ontario and McMaster University Osteoarthritis Index (WOMAC) scores between the 2 groups within 12 weeks postoperatively, and both groups had similar recovery patterns. However, compared to the PA group, the DAA group showed decreased hip flexion and extension strength at 2 and 6 weeks postoperatively.^[16]^ Christensen et al^[33]^ reported no significant differences in Harris Hip Score and timed up-and-go test completion time between the 2 groups at 6 weeks postoperatively. Taunton et al^[31]^ reported an improvement in WOMAC scores in the DAA group compared to the PA group at 3 weeks postoperatively, but no difference at 6 weeks. Poehling-Monaghan et al^[27]^ confirmed no significant difference in daily living activities between the 2 groups postoperatively, with significantly higher Harris Hip Score in the DAA group at 8 weeks postoperatively. Martin et al^[37]^ found that DAA patients had earlier discharge and activity recovery times compared to those undergoing PA during follow-up. Malek et al^[30]^ found no significant difference in Oxford Hip Score between the DAA and PA groups during the 6- to 24-month follow-up period. Rodriguez et al^[40]^ reported that the timed up-and-go test completion time of the DAA group was significantly better than that of the PA group before discharge (*P* < .05) and at 2 weeks postoperatively (*P* < .05). Zhang et al^[15]^ found that at 2 weeks and 1 month postoperatively, the DAA group had significantly higher Harris scores than the PA group (*P* < .05), but there was no statistically significant difference between the 2 groups at 3 and 6 months. Nelms et al^[17]^ found no significant difference in Hip Osteoarthritis Outcome scores between the DAA and PA groups at 1 month postoperatively, but the Hip Osteoarthritis Outcome scores in the DAA group significantly increased after 4 months (*P* < .05).

#### 3.3.2. Postoperative VAS scores

**Figure 3.**
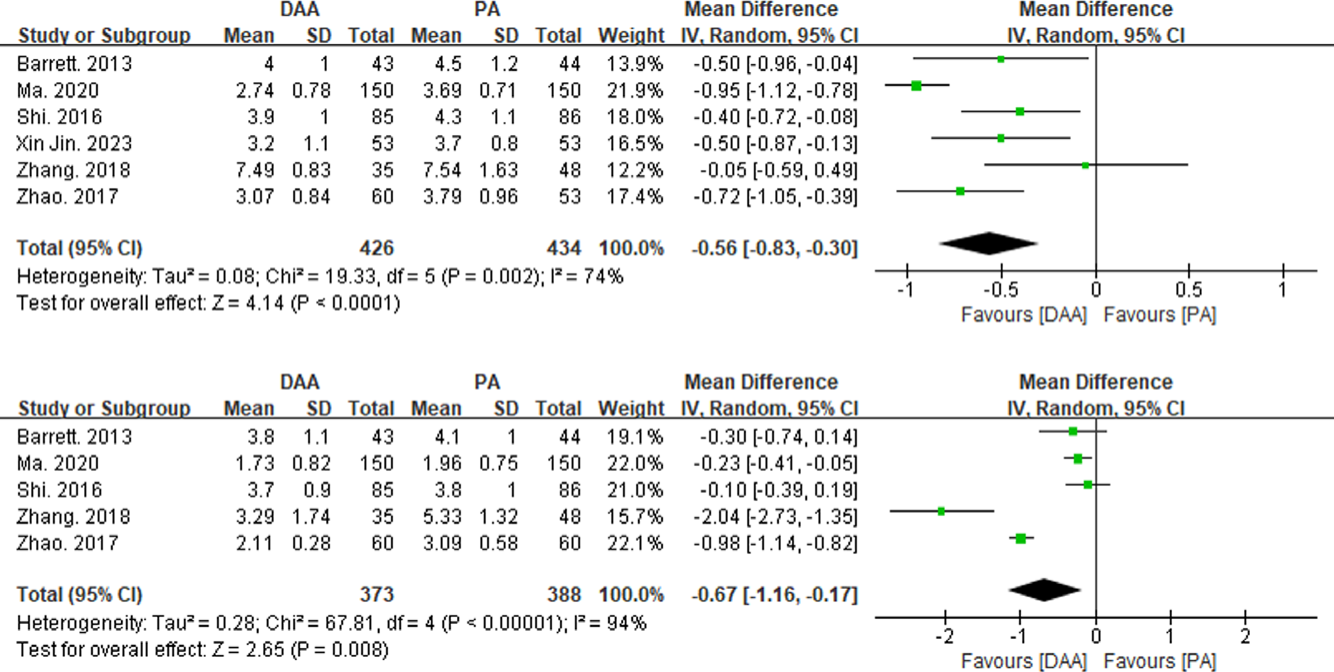
Forest plot of VAS scores comparison between DAA group and PA group on postoperative day 1 and day 2. Six articles reported VAS scores on postoperative day 1 and day 2 (see Fig. 3), and there was significant statistical heterogeneity in VAS scores on postoperative day 1 and day 2. Therefore, a random effects model was used to integrate the MD values of VAS scores on postoperative day 1 and day 2. The meta-analysis results showed that the VAS score of the DAA group on the first day after surgery was significantly higher than that of the PA group (MD = −0.56, 95% CI: −0.83 to 0.30, *P* < .01); The VAS score of the DAA group on the 2nd day after surgery was also significantly higher than that of the PA group (MD = −0.67, 95% CI: −1.16 to 0.17, *P* = .01). CI = confidence interval, DAA = direct anterior approach, MD = mean difference, PA = posterior approach, VAS = visual analogue scale.

Six articles reported VAS scores at postoperative day 1 and postoperative day 2^[15,19,21,29,38,41]^ (see Fig. 3). There was significant statistical heterogeneity in VAS scores at postoperative day 1 and day 2 among the 6 articles. Therefore, a random-effects model was used to integrate the MD values of VAS scores at postoperative day 1 and day 2. The pooled results showed that the VAS scores at postoperative day 1 were significantly lower in the DAA group compared to the PA group (MD = −0.56, 95% CI: −0.83 to −0.30, *P* < .01). Similarly, the VAS scores at postoperative day 2 were also significantly lower in the DAA group compared to the PA group (MD = −0.67, 95% CI: −1.16 to −0.17, *P* = .01).

#### 3.3.3. Intraoperative and postoperative complications

**Figure 4.**
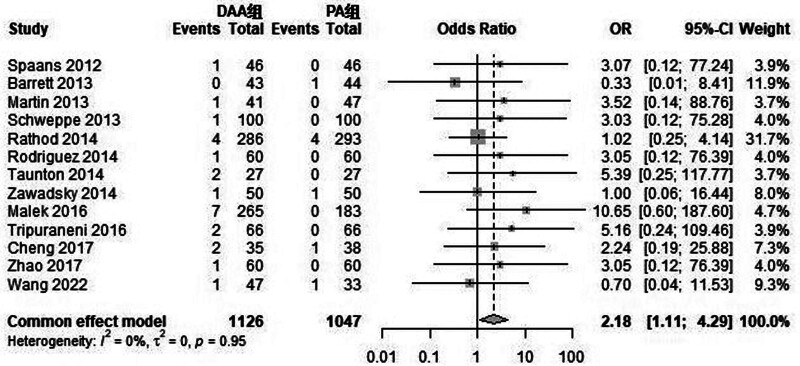
Forest plot comparing intraoperative fractures between DAA group and PA group. There was a significant difference in intraoperative fractures (RR = 2.18, 95% CI: 1.11–4.29, *P* = .05) between the DAA group and the PA group. CI = confidence interval, DAA = direct anterior approach, PA = posterior approach, RR = risk ratio.

**Figure 5.**
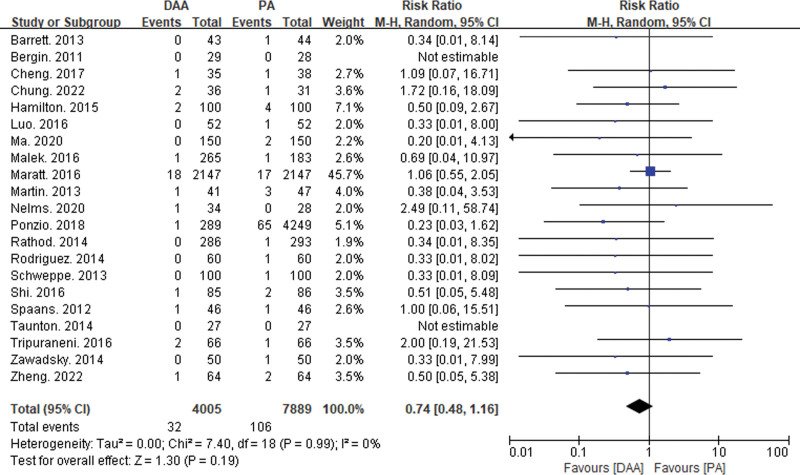
Forest plot of postoperative dislocation comparison between DAA group and PA group. There was no significant difference in postoperative dislocation (RR = 0.74, 95% CI: 0.48–1.16, *P* = .19) between the DAA group and the PA group. CI = confidence interval, DAA = direct anterior approach, PA = posterior approach, RR = risk ratio.

**Figure 6.**
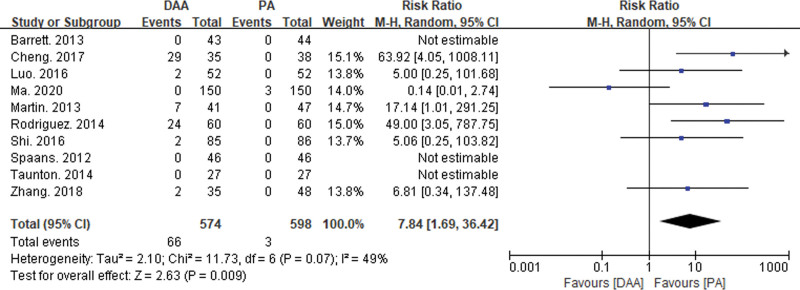
Forest plot comparison of lateral femoral nerve injury between DAA group and PA group. There was a significant difference in lateral femoral nerve injury (RR = 7.84, 95% CI: 1.69–36.42, *P* = .01) between the DAA group and the PA group. CI = confidence interval, DAA = direct anterior approach, PA = posterior approach, RR = risk ratio.

**Figure 7.**
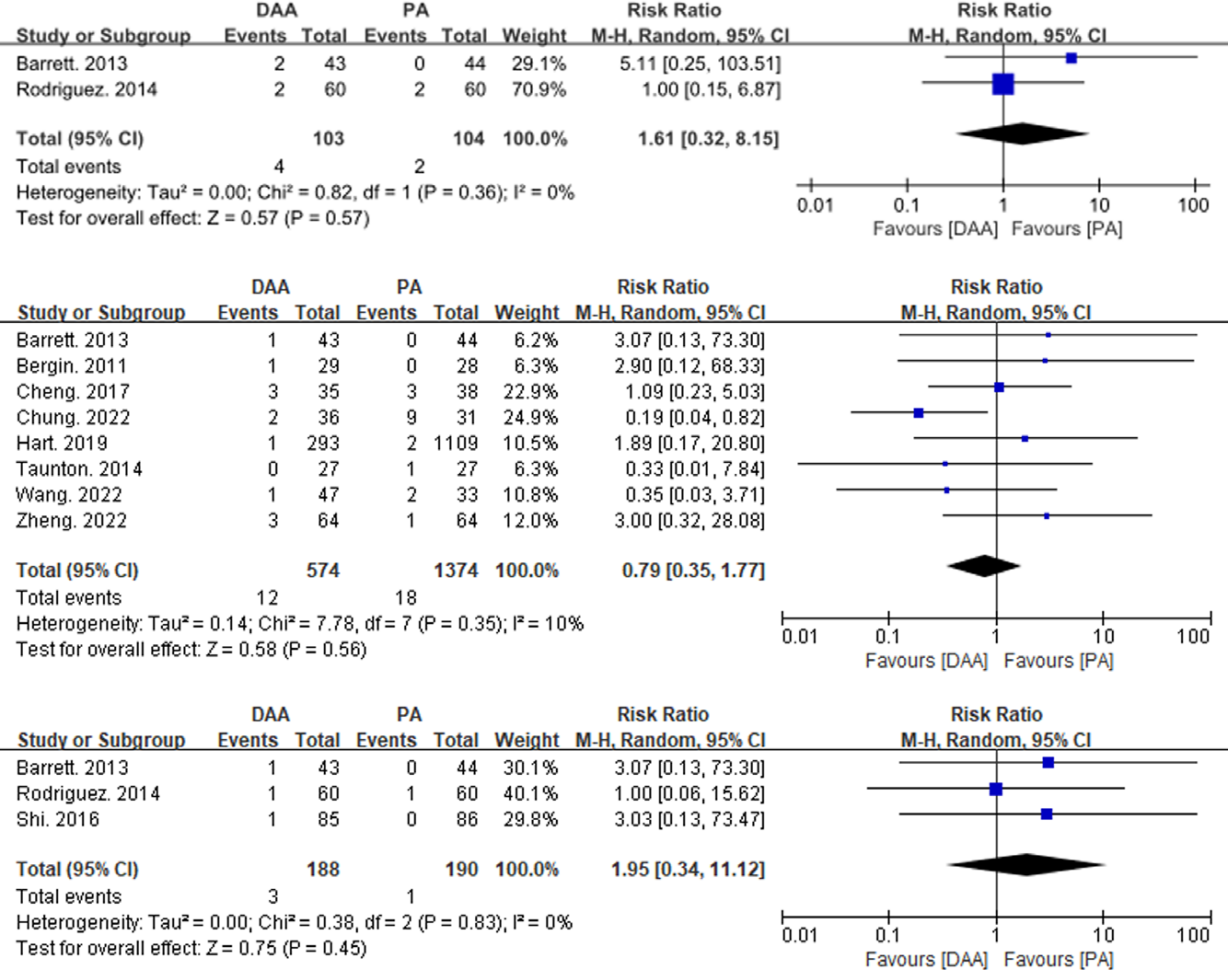
Forest plot comparing pain, incision complications, and ectopic ossification in the inguinal region between the DAA and PA groups. There was no significant difference in inguinal pain (RR = 1.61, 95% CI: 0.32–8.15, *P* = .57), incision complications (RR = 0.73, 95% CI: 0.36–1.50, *P* = .56), and ectopic ossification (RR = 1.95, 95% CI: 0.34–11.12, *P* = .45) between the DAA and PA groups. CI = confidence interval, DAA = direct anterior approach, PA = posterior approach, RR = risk ratio.

This study included 33 articles reporting 6 types of intraoperative and postoperative complications (see Figs. 4, 5, 6, and 7). Intraoperative fractures, postoperative dislocation, groin pain, incision complications, and heterotopic ossification did not exhibit significant statistical heterogeneity. Therefore, a fixed-effects model was used to integrate the RR values for these complications. However, there was significant statistical heterogeneity in femoral nerve injury, so a random-effects model was used to integrate the RR values.

The meta-analysis results showed significant differences between the DAA group and the PA group in intraoperative fractures (RR = 2.18, 95% CI: 1.11–4.29, *P* = .05) and femoral nerve injury (RR = 7.84, 95% CI: 1.69–36.42, *P* = .01). There were no significant differences between the 2 groups in postoperative dislocation (RR = 0.79, 95% CI: 0.35–1.77, *P* = .19), groin pain (RR = 1.61, 95% CI: 0.32–8.15, *P* = .57), incision complications (RR = 0.73, 95% CI: 0.36–1.50, *P* = .56), and heterotopic ossification (RR = 1.95, 95% CI: 0.34–11.12, *P* = .45).

#### 3.3.4. Comparison of operation time, hospital stay, and intraoperative blood loss

**Figure 8.**
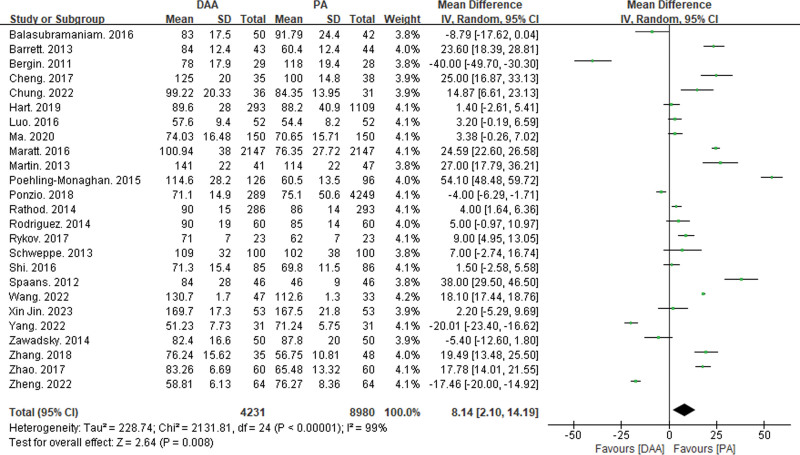
Forest plot of surgical time comparison between DAA group and PA group. There was a significant statistical difference in surgical time (MD = 8.14, 95% CI: 2.1–14.19, *P* < .01) between the DAA and PA groups. CI = confidence interval, DAA = direct anterior approach, MD = mean difference, PA = posterior approach.

**Figure 9.**
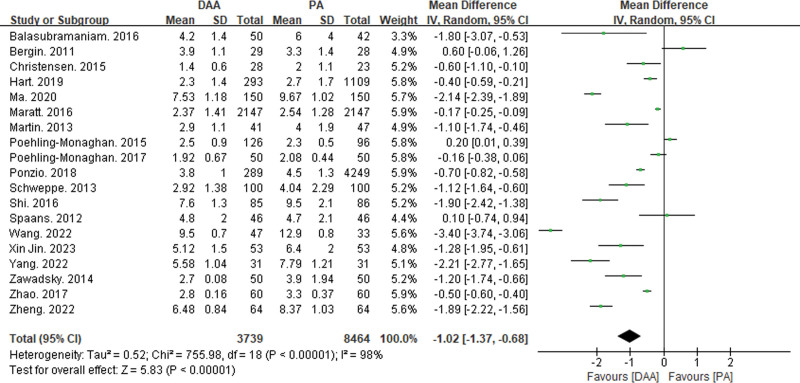
Forest plot of hospitalization time comparison between DAA group and PA group. There was a significant statistical difference in hospitalization time (MD = −1.02, 95% CI: −1.37 to 0.68, *P* < .01) between the DAA and PA groups. CI = confidence interval, DAA = direct anterior approach, MD = mean difference, PA = posterior approach.

**Figure 10.**
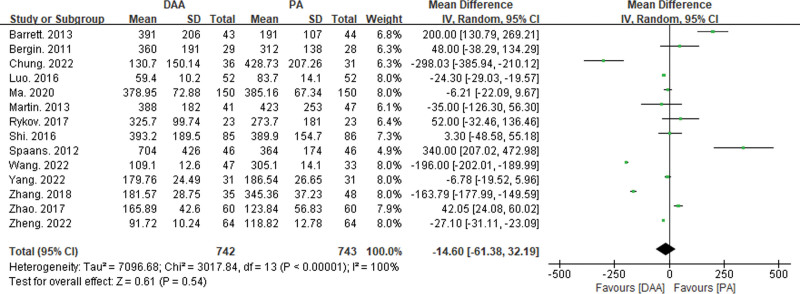
Forest plot of intraoperative blood loss comparison between DAA group and PA group. There was no significant statistical difference in intraoperative bleeding volume (MD = −14.6, 95% CI: −61.38 to 32.19, *P* = .54) between the DAA and PA groups. CI = confidence interval, DAA = direct anterior approach, MD = mean difference, PA = posterior approach.

Twenty-five articles reported on operation time^[15,16,18–21,23–25,27–29,32,34,35,37–39,42]^ (Fig. 8), 19 articles reported on hospital stay^[19–21,23,25,27,28,32,33,35,37–39,41–46]^ (Fig. 9), and 14 articles reported on intraoperative blood loss^[15,18–21,29,32,34,37,38,43–45,47]^ (Fig. 10). There was significant statistical heterogeneity in operation time, hospital stay, and intraoperative blood loss, so a random-effects model was used to integrate the MDs.

The meta-analysis results showed significant statistical differences between the DAA and PA groups in operation time (MD = 8.14, 95% CI: 2.1–14.19, *P* < .01) and hospital stay (MD = −1,02, 95% CI: −1.37 to −0.68, *P* < .01). However, there was no significant statistical difference in intraoperative blood loss between the DAA and PA groups (MD = −14.6, 95% CI: −61.38 to 32.19, *P* = .54).

#### 3.3.5. Comparison of postoperative imaging results

**Figure 11.**
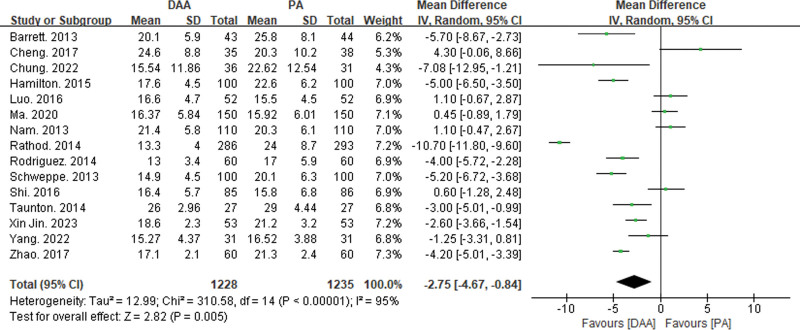
Forest plot of acetabular anteversion angle comparison between DAA group and PA group. There is a significant statistical difference in acetabular anteversion angle (MD = −2.75, 95% CI: −4.67 to 0.84, *P* < .01) between the DAA and PA groups. CI = confidence interval, DAA = direct anterior approach, MD = mean difference, PA = posterior approach.

**Figure 12.**
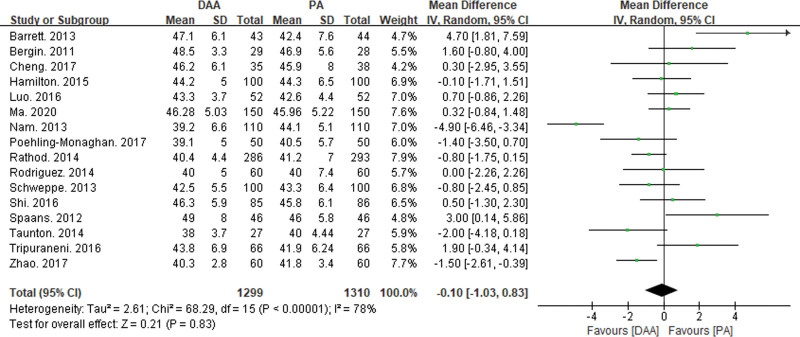
Forest plot of acetabular abduction angle comparison between DAA group and PA group. There was no significant statistical difference in acetabular abduction angle (MD = −0.10, 95% CI: −1.03 to 0.83, *P* = .83) between the DAA and PA groups. CI = confidence interval, DAA = direct anterior approach, MD = mean difference, PA = posterior approach.

Fifteen articles reported on acetabular anteversion angle^[16,19,21–24,26,29,31,34,38,40,43,46,47]^ (see Fig. 11), and fifteen articles reported on acetabular abduction angle^[16,19–23,26,27,29,31,32,34,36,38,40,43,46,47]^ (Fig. 12). Both parameters exhibited significant statistical heterogeneity, so a random-effects model was used to integrate the MD values.

The meta-analysis results showed a significant statistical difference in acetabular anteversion angle (MD = −2.75, 95% CI: −4.67 to −0.84, *P* < .01) between the DAA and PA groups. However, there was no significant statistical difference in acetabular abduction angle (MD = −0.10, 95% CI: −1.03 to 0.83, *P* = .83) between the DAA and PA groups.

**Figure 13.**
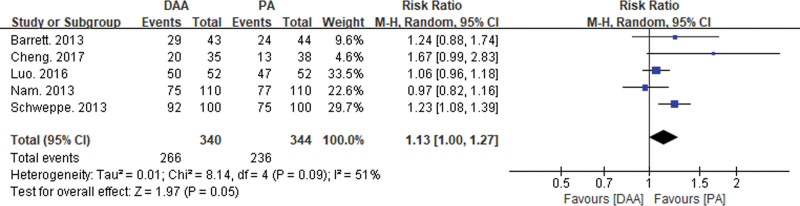
Forest plot comparing the number of acetabular implants in the Lewinnek safe zone between the DAA and PA groups. There are 5 literature reports on the number of Lewinnek safe zone acetabular implants,^[13,18,19,29,31]^ and there is significant statistical heterogeneity. The random effects model showed a significant statistical difference in the number of acetabular implants in the Lewinnek safe zone (RR = 1.13, 95% CI: 1.00–1.27, *P* = .05) between the DAA and PA groups. CI = confidence interval, DAA = direct anterior approach, PA = posterior approach, RR = risk ratio.

Five articles reported on the number of prostheses placed within the Lewinnek safe zone^[16,22,23,29,34]^ (see Fig. 13), and there was significant statistical heterogeneity. The random-effects model showed a significant statistical difference in the number of prostheses within the Lewinnek safe zone (RR = 1.13, 95% CI: 1.00–1.27, *P* = .05) between the DAA and PA groups.

## 4. Discussion

This study incorporated the latest clinical research and compared it with previously published meta-analyses. A total of 33 articles were included, comprising 14,478 participants, with 4911 in the DAA group and 9567 in the PA group, to compare the postoperative clinical efficacy of DAA and PA. The results of this study showed that the VAS scores on postoperative day 1 (MD = −0.57, 95% CI: −0.82 to −0.33, *P* < .01) and day 2 (MD = −0.67, 95% CI: −1.16 to −0.17, *P* = .01) were significantly lower in the DAA group compared to the PA group. The DAA group had a significantly higher risk of intraoperative fractures (RR = 2.18, 95% CI: 1.11–4.29, *P* = .05) and lateral femoral cutaneous nerve injury (RR = 10.96, 95% CI: 1.72–69.83, *P* = .01) compared to the PA group. The operative time was significantly longer in the DAA group (MD = 8.16, 95% CI: 0.59–15.72, *P* < .01), while the hospital stay was significantly shorter (MD = −1.03, 95% CI: −1.49 to −0.57, *P* < .01). The acetabular anteversion angle was significantly smaller in the DAA group compared to the PA group (MD = −2.75, 95% CI: −4.64 to −0.86, *P* < .01), and the number of prostheses placed in the Lewinnek safe zone was significantly higher in the DAA group (RR = 1.93, 95% CI: 1.06–3.52, *P* = .03). There were no significant differences between the DAA and PA groups in terms of postoperative cessation of walking aids, postoperative dislocation, groin pain, incision complications, heterotopic ossification, and intraoperative blood loss (*P* > .05).

This study found that patients undergoing DAA surgery had better postoperative recovery compared to those undergoing PA surgery. Specifically, the DAA group showed superior relief of postoperative pain symptoms, with lower VAS scores and WOMAC scores.^[16]^ Moreover, fewer patients in the DAA group required walking aids and analgesics within 6 weeks postoperatively.^[25]^ The differences in postoperative recovery were attributed to the surgical approach, with DAA causing less damage to surrounding tissues compared to PA.^[48,49]^ The differences in surgical procedures directly affected postoperative symptom relief and functional recovery.^[44]^ Additionally, factors such as intraoperative pain management, surgeon experience, and postoperative rehabilitation training were noted to influence postoperative symptom relief and functional recovery.

The study also observed a significantly shorter average hospital stay in the DAA group, confirming that DAA causes less damage to surrounding tissues and leads to faster postoperative recovery.

The placement of the prosthesis in the acetabulum is crucial in THA, impacting postoperative functional recovery, complications, and prosthesis longevity. The study found a significantly higher number of prostheses placed in the Lewinnek safe zone in the DAA group, attributed to the use of X-ray fluoroscopy during surgery. However, this came with the trade-off of higher ionizing radiation exposure for patients in the DAA group.

Regarding complications, the study identified a higher risk of intraoperative fractures and lateral femoral cutaneous nerve injury in the DAA group, along with a significantly longer operative time. This is related to the surgeon’s experience and learning curve. Previous studies have found that there is no significant difference in the incidence of postoperative complications between the DAA group and the PA group after passing through the learning curve.^[29,31,40]^ Similarly, due to being in the learning curve, the incidence of complications in the DAA group is significantly higher than that in the PA group.^[20,50]^ This indicates that the postoperative complications and surgical time of THA are greatly influenced by the surgeon’s experience, and more careful surgical procedures are needed to effectively avoid postoperative complications for patients.

The main disadvantage of DAA is that it is more prone to intraoperative complications, which can be greatly improved by the application of X-ray fluoroscopy for positioning and robot assisted surgery. Therefore, this disadvantage is expected to be effectively compensated for in the future. This means that with the promotion of new orthopedic technologies, DAA surgery may have broader application prospects in the future.

Previous comparative studies have generally favored DAA, with some reporting higher levels of creatine kinase in the PA group and improved hip joint mobility in the DAA group.^[24,48]^ However, some studies found no significant differences in quadriceps strength between the 2 groups.^[51]^ Additionally, PA was associated with a lower risk of heterotopic ossification compared to DAA.^[52]^

Compared to previous meta-analyses comparing direct anterior and PAs for THA, this study included more literature and a larger sample size to conduct the most comprehensive clinical outcome evaluation. Our analysis included all important outcome indicators used and not used in previous studies, using 17 indicators to comprehensively compare the differences in anatomy, imaging, function, complications, pain, and recovery speed between the 2 surgical methods during and after surgery, effectively avoiding the one-sided conclusion caused by ignoring important indicators. We have significant advantages in evidence strength, comprehensiveness, and persuasiveness of the comparison. In terms of results, we have for the first time confirmed the advantage of DAA over PA in surgical anatomy, which means that patients who choose DAA approach tend to have better hip joint stability after surgery.

The study acknowledges several limitations, including the predominance of non-randomized controlled trials, inconsistencies in follow-up time and assessment indicators across studies, variations in patient characteristics, and the lack of data on the evaluation of soft tissues around the hip joint. The authors call for future research to address these limitations through rigorously designed randomized controlled trials.

## 5. Conclusion

This study suggests that patients in the DAA group exhibit better postoperative recovery, shorter hospital stays, and improved functional recovery and pain relief compared to the PA group. Additionally, the DAA group demonstrates a significantly higher number of prostheses placed in the Lewinnek safe zone.

## Author contributions

**Conceptualization:** Hao Wang, Jin-Feng Liu.

**Data curation:** Hao Wang, Tiange Yuan, Hengduo Jiang.

**Formal analysis:** Hao Wang, Tiange Yuan, Zhuoqi Wei.

**Funding acquisition:** Jin-Feng Liu.

**Investigation:** Fengjing Wang, Hengduo Jiang, Yang Zhang, Jiahao Meng.

**Methodology:** Hengduo Jiang, Jiahao Meng.

**Resources:** Hengduo Jiang.

**Software:** Tiange Yuan, Hengduo Jiang, Zhuoqi Wei.

**Supervision:** Jin-Feng Liu, Jiahao Meng.

**Validation:** Fengjing Wang.

**Visualization:** Jin-Feng Liu.

**Writing – original draft:** Hao Wang.

**Writing – review & editing:** Jin-Feng Liu, Jiahao Meng.
